# An Explorative Study on Monocyte Reprogramming in the Context of Periodontitis *In Vitro* and *In Vivo*

**DOI:** 10.3389/fimmu.2021.695227

**Published:** 2021-08-13

**Authors:** Marlies P. Noz, Adelina S. Plachokova, Esther M.M. Smeets, Erik H. J. G. Aarntzen, Siroon Bekkering, Prya Vart, Leo A. B. Joosten, Mihai G. Netea, Niels P. Riksen

**Affiliations:** ^1^Department of Internal Medicine and Radboud Institute for Molecular Life Science (RIMLS), Radboud University Medical Center, Nijmegen, Netherlands; ^2^Department of Dentistry, Radboud University Medical Center, Nijmegen, Netherlands; ^3^Department of Radiology, Nuclear Medicine and Anatomy, Radboud University Medical Center, Nijmegen, Netherlands; ^4^Department of Health Evidence and Radboud Institute for Health Sciences (RIHS), Radboud University Medical Center, Nijmegen, Netherlands; ^5^Department of Medical Genetics, Iuliu Hatieganu University of Medicine and Pharmacy, Cluj-Napoca, Romania; ^6^Department for Genomics & Immunoregulation, Life and Medical Sciences Institute (LIMES), University of Bonn, Bonn, Germany

**Keywords:** periodontitis, *Porphyromonas gingivalis*, trained immunity, inflammation, cardiovascular disease

## Abstract

**Aims:**

Periodontitis is an independent risk factor for cardiovascular disease, but the mechanistic link is not fully understood. In atherosclerotic cardiovascular disease, monocytes can adopt a persistent hyperresponsive phenotype, termed trained immunity. We hypothesized that periodontitis-associated bacteria can induce trained immunity in monocytes, which subsequently accelerate atherosclerosis development.

**Materials and Methods:**

We combined *in vitro* experiments on human primary monocytes and *in vivo* techniques in patients with periodontitis to test this hypothesis. Adherent peripheral blood mononuclear cells (PBMCs) were transiently exposed *in vitro* to *Porphyromonas gingivalis* for 24 hours, and restimulated with lipopolysaccharide (LPS) or Pam3CysK4 (P3C) six days later, to measure interleukin-6 (IL-6) and tumor necrosis factor α (TNFα) production. In an exploratory observational study, patients with severe periodontitis (63 ± 6 years, n=14) and control subjects with no-to-mild periodontitis (54 ± 10 years, n=14) underwent venipuncture and 2’-deoxy-2’-[^18^F]fluoro-D-glucose positron-emission-tomography ([^18^F]FDG PET/CT) scanning.

**Results:**

When adherent peripheral blood mononuclear cells (PBMCs) were transiently exposed *in vitro* to *Porphyromonas gingivalis* for 24 hours, and restimulated with LPS or P3C six days later, IL-6 and TNFα production was significantly increased (TNFα/P3C, p<0.01). Circulating leukocytes, IL-6 and interleukin-1 receptor antagonist (IL-1Ra) concentrations were generally higher in patients compared to controls (leukocytes: p<0.01; IL-6: p=0.08; IL-1Ra: p=0.10). Cytokine production capacity in PBMCs after 24h stimulation revealed no differences between groups. [^18^F]FDG PET/CT imaging showed a trend for increased [^18^F]FDG-uptake in the periodontium [mean standard uptake value (SUV_mean_), p=0.11] and in femur bone marrow (SUV_mean_, p=0.06), but no differences were observed for vascular inflammation. Positive correlations between severity of periodontitis, measured by The Dutch Periodontal Screening Index and pocket depth, with circulating inflammatory markers and tissue inflammation were found.

**Conclusions:**

*P. gingivalis* induces long-term activation of human monocytes *in vitro* (trained immunity). Patients with severe periodontitis did have signs of increased systemic inflammation and hematopoietic tissue activation. However, their circulating monocytes did not show a hyperresponsive phenotype. Together we suggest that trained immunity might contribute to local periodontal inflammation which warrants further investigation.

## Introduction

Periodontitis (PD) is an independent risk factor for cardiovascular disease (CVD) (1, 2). Unravelling the mechanistic link between PD and CVD is of great public health importance due to their high prevalence and economic burden (3, 4). Despite ongoing research, a comprehensive understanding of this association is still lacking (5).

PD and CVD are chronic low-grade inflammatory diseases, driven by the activation of innate immune cells. In atherosclerosis, which is the main cause of CVD, monocytes and macrophages play a key role in the initiation, progression, and destabilization of atherosclerotic plaques (6). In PD, microbial dysbiosis and local inflammation co-develop in a reciprocally reinforced manner (7). *Porphyromonas gingivalis* is a keystone periodontal pathogen driving microbial dysbiosis in PD (8, 9), and is also suggested to be involved in atherosclerotic CVD. It was isolated from human atherosclerotic plaques (1), and reported to modulate immune cell function (10). In mice, *P. gingivalis*-induced PD led to atherosclerosis with enhanced vascular wall inflammation (11). We now propose that a novel mechanism of innate immune cell activation, called trained immunity, contributes to the association between PD and CVD.

Trained immunity describes the observation that monocytes/macrophages can build immunological memory after encountering a pathogen, resulting in a persistent hyperresponsive phenotype. Increased cytokine production capacity is the hallmark of trained immunity. It provides host protection against recurrent infections, but in chronic inflammatory diseases, such as atherosclerosis, it can contribute to the disease pathophysiology (12). Trained immunity can be induced by brief exposure to micro-organisms, but also with endogenous atherogenic stimuli. In human monocytes, exposure to oxidized low-density lipoprotein (oxLDL) induces a persistently enhanced cytokine response and increased foam cell formation (13). Trained immunity develops through distinct epigenetic and metabolic reprogramming (12).

In view of the association between PD and CVD, we now hypothesize that *P. gingivalis* as a keystone periodontal pathogen can induce trained immunity *in vitro*, and that monocytes isolated from patients with PD have a hyperresponsive phenotype that accelerates atherosclerosis development.

## Methods

### *In Vitro* Methods

#### Culturing *P. gingivalis*

*P. gingivalis* strains ATCC 33277 and W83 (kindly provided by ACTA, Amsterdam, The Netherlands) were cultured on blood agar plates under anaerobic conditions at 37°C. At day 7, colonies were transferred to anaerobe basal broth (CM0957, ThermoFisher, Waltham, USA) and cultured for 24h until they reached mid-log growth. Gram staining confirmed purity of the cultures. The bacterial cultures (OD_690_ 0.1) were heat-inactivated at 60°C for 60min.

#### PBMC Isolation

Buffy coats were obtained from healthy donors after written consent (Sanquin Blood Bank, Nijmegen, the Netherlands). Peripheral blood mononuclear cells (PBMCs) were isolated using density centrifugation over Ficoll-Paque PLUS (GE Healthcare Biosciences, Chicago, USA). Cells were resuspended in Dutch modified RPMI culture medium (Invitrogen, CA, USA) supplemented with 2mmol/L glutamine, 10mg/mL gentamicin and 1mmol/L pyruvate.

#### Trained Immunity Model

A validated trained immunity experimental design was used (14, 15). After 1h incubation with 5x10^5^ PBMCs per well, cells were washed thrice to remove non-adherent cells in flat-bottom 96-well plates (Corning, NY, USA). Adherent monocytes were stimulated with 1x10^4^/mL *P. gingivalis* W83, 1x10^4^/mL *P. gingivalis* ATCC, or RPMI only. After 24h incubation, the cells were washed with warm PBS and incubated in culture medium with 10% pooled human serum. At day 6, cells were restimulated with RPMI, 10ng/mL *Escherichia coli* lipopolysaccharide (LPS; serotype 055:B5 Sigma-Aldrich, St. Louis, USA), and 10μg/mL Pam3CysK4 (P3C; EMC Microcollections, Tübingen, Germany). At day 7, supernatants were collected and stored at −20°C.

In separate experiments, *P. gingivalis* stimulation was combined with freshly oxidized LDL [10µg/mL, prepared as described previously (16)] for 24h in adherent monocytes. In brief, LDL was isolated from healthy volunteers under sterile conditions and stored at -80 with saccharose and EDTA to prevent oxidation. Upon thawing, LDL was dialyzed with sterile PBS for 7h and oxidated overnight with filter sterilized copper sulfate. Contamination by endotoxins has been previously ruled out by the original protocol (13, 16). After 24h stimulation, the stimuli were washed away and the similar protocol as noted above was followed.

#### Cytotoxicity

Cytotoxicity was determined after 24h of incubation with *P. gingivalis* using a lactate dehydrogenase cytotoxicity assay (LDH; CytoTox-96-Non-Radioactive-Cytotoxicity-Assay, Promega, USA) on fresh supernatants.

#### Foam Cell Formation

Foam cell formation was evaluated after 4h starvation and 24h oxLDL (25µg/mL) exposure at day 6 of the trained immunity experiment. On day 7, Oil Red O staining was performed on adherent macrophages. Intracellular ApoB concentration was measured using enzyme-linked immunosorbent assay (ELISA) as described in (17).

#### Cytokine Measurements

Cytokine production in supernatants was determined using DuoSet ELISA for TNFα and IL-6 according to the manufacturers’ instructions (R&D Systems, Minneapolis, USA).

### *In Vivo* Methods

#### Study Participants

Participants (40-80 years) were recruited among patients of the Dentistry department of Radboud University Medical Center, the Netherlands from 03-2018 till 02-2019. At the department of Dentistry standard dental care is provided and the patients represent the average population in the area, *i.e.* they are not referred because of dental problems. Exclusion criteria were previous CVD, auto-immune or auto-inflammatory diseases (including diabetes mellitus), chronic immunomodulatory drug use, chronic kidney disease (MDRD<45ml/min) or liver disease (ALAT>135U/l). Additionally, participants with an infection (>38,5°C or antibiotic treatment), hospital admission, or vaccination within 1-month prior study entry were excluded. We attempted to match control participants based on age, sex, body mass index (BMI) and smoking. The study protocol was approved by the Institutional Review Board Arnhem/Nijmegen, the Netherlands (NL61840.091.17) and conform STROBE Guidelines. All participants gave written informed consent.

#### Clinical Periodontal Assessment

The Dutch Periodontal Screening Index (DPSI) and probing pocket depth (PPD) were recorded by an experienced periodontist [AP]. Subjects with DPSI scores 0-2 were classified as having no PD (category A), those with DPSI score 3- as having mild PD (category B), and subjects with DPSI scores 3+ and 4 as having severe PD (category C) (18). The presence of alveolar bone loss on paired bitewings, defined as >2 mm distance between the cement-enamel junction and alveolar bone crest (19) was used to confirm the clinical diagnosis. Subjects with DPSI category C and advanced alveolar bone loss were assigned to the patient group, *i.e.* participants with severe PD. Subjects with DPSI category A and B without radiographic alveolar bone loss were assigned to the control group, *i.e.* participants with no-to-mild PD, which is representative for the normal population in this age category.

Defined by the New Periodontal classification of 2018 (20), patients had PD-stage II-IV, whilst controls had gingivitis or PD-stage I.

#### Cardiovascular Risk Assessment

Blood pressure was measured according to AHA guidelines (21). Fasting total cholesterol (Tchol), high-density lipoprotein cholesterol (HDLc), and triglycerides (TG) were measured using standardized methods, and LDL cholesterol (LDLc) was calculated with the Friedewald formula.

#### PBMC Isolation and Stimulation

PBMCs were isolated by Ficoll-Paque density gradient centrifugation (GE Healthcare) and resuspended in supplemented RPMI. Cell composition was evaluated by Sysmex analyzer (Sysmex). Per well, 5x10^5^ PBMCs were stimulated for 24h in triplicate in round-bottom 96-well plates (Corning) with the following stimuli to assess the innate immune response: RPMI, 10ng/mL LPS and 10μg/mL P3C. Simultaneously, to assess the adaptive immune response, PBMCs were stimulated in duplicate for 7days with RPMI, 1×10^6^/mL *Candida albicans conidia* (UC820 strain), or 1x10^6^/mL *Staphylococcus aureus* (ATCC29213 strain) both with 10% human pool serum. After the incubation periods of 24h and 7days, supernatants were stored after plate centrifugation at −80°C.

#### Cytokine Measurements

Cytokine and chemokine concentrations were determined in supernatants using DuoSet ELISA for TNFα, IL-6, IL-8, IL-10, IL-1β, IL-17 and IL-22 (R&D), and interferon gamma (IFN-γ) (Sanquin). Plasma hsCRP concentrations were obtained (R&D). Circulating IL-1β, IL-1Ra, IL-6 and IL-18 concentrations were sensitively measured using SimplePlex cartridge on the Ella platform (Protein Simple, San Jose, USA).

#### Flow Cytometry

Circulating immune cells and monocyte subpopulations were determined by their expression markers using a CYTOflex flow cytometer (Beckman Coulter). 50 µL EDTA blood was stained after the lysis-no-wash strategy (BD Pharm Lyse lysing buffer, Becton Dickinson) by monoclonal antibodies CD45 Krome Orange ([KO], clone J33; Beckman Coulter), HLA-DR PE (clone immu-357; Beckman Coulter), CD14 PC7 (clone 61D3 Bioscience), CD16 FITC (clone CB16; eBioscience), CD3 APC-Alexa750 (clone UCTH1; Beckman Coulter), CD56 APC (clone N901; Beckman Coulter), CD192 Brilliant Violet421 ([BV421] clone 48607; Becton Dickinson), CD11b BV785 (clone ICRF44; Biolegend), CD41 PC5.5 (clone Hip8; Biolegend) and measured with CytoFLEX flow cytometer (Beckman Coulter).

Single cells were analyzed by manual gating using Kaluza v2.1 software (Beckman Coulter) and unsupervised computational methods in parallel using FlowJo v10.6.2 software (Beckton Dickinson). The gating strategy applied is shown in **Appendix Figure 1**, gates were set with the fluorescence-minus-one method (22, 23). In short, monocytes were selected based on CD45+ HLA-DR+ and monocyte scatter properties, then CD3+ T-lymphocytes and CD56+ NK-cells were excluded, and monocyte subsets were identified in the CD14/CD16 plot as percentage of gated. Data was analyzed with Kaluza 2.1 software (Beckman Coulter). Characterization of monocytes subsets is according to current recommendations (22, 23).

For unsupervised computation analyses, data files were randomly down sampled to 10,000 events per file and subsequently concatenated to a single file containing all events. Controls and patients were labelled accordingly to allow separation after analysis. Unsupervised clustering was performed on the expression values of all markers using the tSNE plugin in FlowJo v10.6.2 software (Becton Dickinson), using 300 iterations and a perplexity of 20. Manual gating of cell populations (based on gating strategy) was used to identify cell populations and check the separation quality of the unsupervised clustering. The contribution of periodontitis to circulating cell populations in the total tSNE was inspected by separating the patient and control group in two figures (**Figure 3**). Visual differences were confirmed by manual gating and statistical analysis.

#### [^18^F]FDG PET and Low-Dose CT Scanning

After adhering to a 24h low-carbohydrate diet and 6h of fasting, participants underwent 2’-deoxy-2’-[^18^F]fluoro-D-glucose positron-emission-tomography ([^18^F]FDG PET) with low-dose non-contrast enhanced CT on a dedicated Siemens Biograph 40mCT scanner (Siemens Healthineers, Erlangen, Germany). Analyses were performed on reconstruction settings according to European guidelines (24) using Inveon Research Workspace v4.2 software. See supplement for details.

Periodontal [^18^F]FDG-uptake was determined in the left and right periodontium of maxilla-to-mandible (**Figure 4A**, **Appendix Figure 2**). Vascular [^18^F]FDG-uptake was determined in the aorta ascendens, aorta descendens, abdominal aorta, the left and right common carotid arteries, and the left and right iliac arteries (**Figure 4B**, illustration). Hematopoietic [^18^F]FDG-uptake was assessed in the spleen, lumbar vertebrae L2/L3, and the left and right medullary femur cavity (**Figure 4C**, illustration). The mean standardized uptake value (SUV_mean_) was calculated after correction for weight and [^18^F]FDG-dose (MBq). The target-to-background ratio (TBR) was calculated as the ratio of vascular wall SUV_mean_ and arterial bloodpool SUV_mean_. Hematopoietic tissue TBR was expressed as ratio of liver SUV_mean_.

#### Statistical Analysis

*In vitro* data are reported as mean ± SEM. *P. gingivalis*-trained cells were compared to RPMI-incubated control cells using Wilcoxon signed-rank test. The *in vivo* study is exploratory, hence no sample size calculation is performed. *In vivo* continuous variables are reported as mean ± SD or median[interquartile range], depending on the data distribution. Normal distribution of the data was checked with the Shapiro-Wilk test, when the p-value reached <0.05 this assumption was violated. Patients and controls were compared for continuous variables using independent samples T-test (if normally distributed) or Mann-Whitney U test (if not normally distributed). Categorical variables are presented as percentage(number) and were compared using Χ^2^-test. For all study outcomes, a one-way ANCOVA was conducted to control for age differences between patients and controls. Beforehand, outliers were removed with > ± 3SD of Z-scores and thereafter log(10)-transformed. Missing values were not imputed. A two-sided p-value <0.05 was considered statistically significant. All data were analyzed using SPSS v25.0 (Chicago, USA).

## Results

### *P. gingivalis* Induces Trained Immunity *In Vitro*

**Figure 1:**
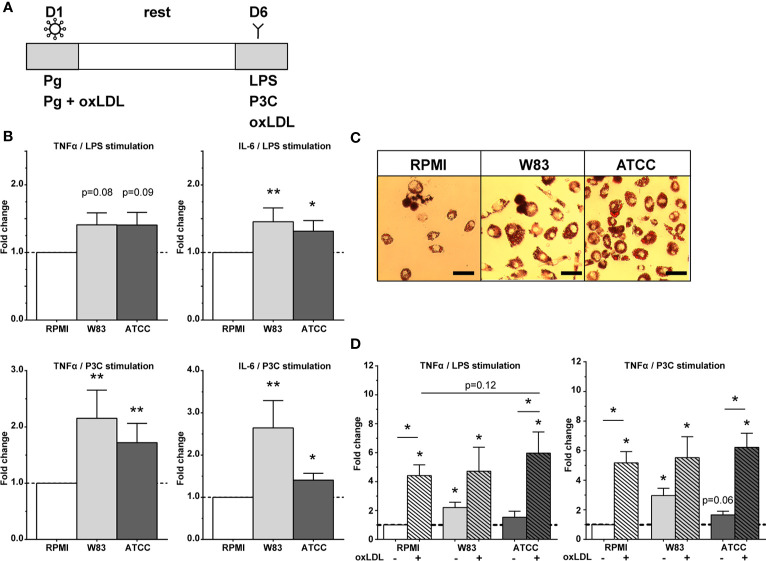
Trained immunity by *P. gingivalis*. **(A)** Overview of trained immunity model. Adherent monocytes were trained for 24h on day 1 with Pg or Pg with oxLDL (10µg/mL). After 24h the training stimulus was washed away, the cells were rested for 6 days in supplemented RPMI after which the cells were restimulated on day 6 with RPMI, LPS, or P3C for 24h (without oxLDL) to measure cytokine production, or they were exposed to oxLDL (25 µg/mL) for 24 hours to induce foam cell formation. On day 7, TNFα and IL-6 concentrations were measured in the supernatants. **(B)** *P. gingivalis* induces trained immunity as shown by the increased fold change of TNFα and IL-6 (n=20, *p < 0.05, **p < 0.01). **(C)** Foam cell formation by *P. gingivalis* using Oil Red O staining. Magnification x40, scale bar 50µm (n=6, representative picture is shown). **(D)** *P. gingivalis* in combination with oxLDL exposure for 24h on day 1 increased training response (n = 6, *p < 0.05). Data are presented as fold change with mean ± SEM. *P. gingivalis*-trained cells were compared to RPMI-incubated control cells at day 1 after restimulation with LPS or P3C at day 6. Wilcoxon signed-rank test. Pg, *P. gingivalis*; oxLDL, oxidized LDL; W83, Pg W83 strain; ATCC, Pg ATCC strain; RPMI, medium control.

The *in vitro* experiments with *P. gingivalis* ATCC and W83 strains are illustrated in **Figure 1A**. The production of IL-6 and TNFα after restimulation was significantly increased compared to the RPMI-incubated control cells (**Figure 1B**, noted in fold change p<0.05). Restimulation with P3C, a TLR2 agonist, resulted in the strongest augmentation of the cytokine response with a two-fold induction. The absolute cytokine production is shown is **Appendix Table 1**.

In *P. gingivalis*-trained macrophages, foam cell formation seemed to be increased as visualized by Oil Red O staining (**Figure 1C**). Quantification of the intracellular lipid uptake (ApoB) did not show differences (p=0.20, data not shown).

**Figure 2:**
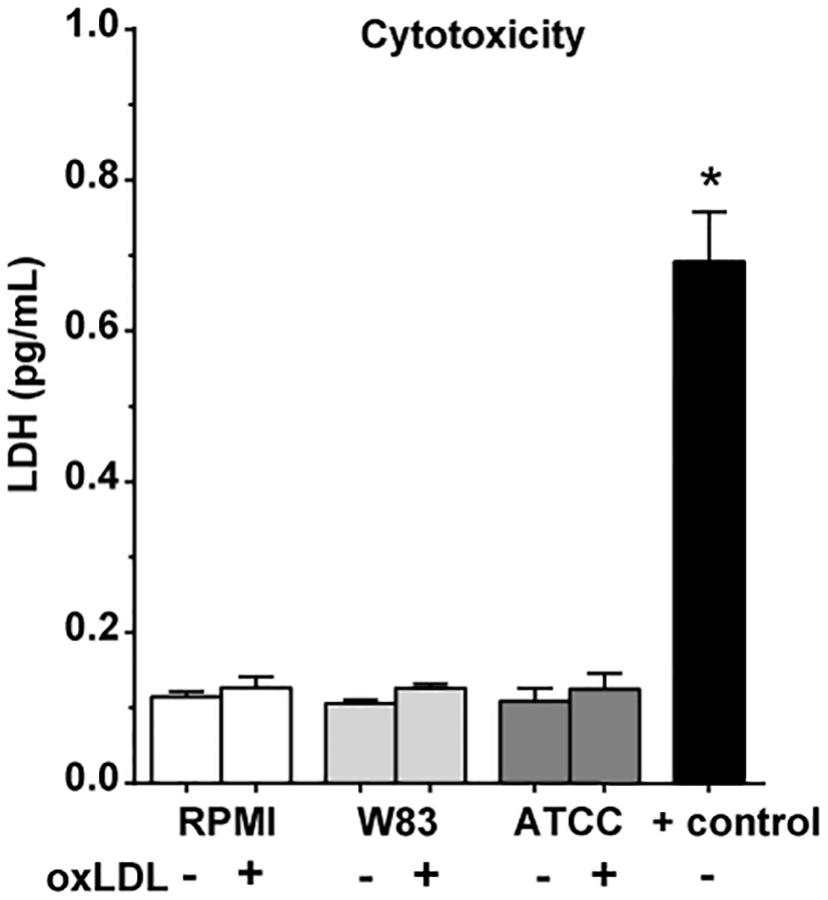
Cytotoxicity of stimuli. Cell viability after 24h *P. gingivalis* and oxLDL costimulation (n = 6, *p < 0.05) measured with LDH cytotoxicity assay. Data are presented with mean ± SEM, p-values using Wilcoxon signed-rank test. oxLDL, oxidized LDL; W83, Pg W83 strain; ATCC, Pg ATCC strain; RPMI, medium control; + control, positive control for cell death.

Both oxLDL and *P. gingivalis* can be found in low concentrations in the periodontium (25). Costimulation with *P. gingivalis* and oxLDL in the first 24h resulted in a strong amplification of cytokine production upon restimulation compared to the RPMI-exposed control. The induction of trained immunity by combined stimulation was not stronger compared to oxLDL training alone, although there was a trend for ATCC to further potentiate LPS-induced TNFα release (**Figure 1D**, p=0.12). Costimulation did not influence cell viability (**Figure 2**).

### Patients With Severe Periodontitis Have Increased Systemic Inflammation Which Correlates With Tissue Inflammation

#### Baseline Characteristics

We included participants with severe PD (DPSI 3.9 ± 0.2, n=14, ‘patients’) and participants with no-to-mild PD (DPSI 2.9 ± 0.3, n=14, ‘controls’), which is a representative of the general population (**Table 1**). Patients were older than controls (mean 63 ± 6 *versus* 54 ± 10 years, p<0.05), but there was no significant different in classical cardiovascular risk factors. Patients only had a higher previous tobacco exposure (15.8 ± 18.2 *versus* 4.5 ± 5.7 pack years, p<0.05), but current smoking behavior was similar. Age is known to impact immune cell function (26) and future cardiovascular risk, therefore we corrected all comparisons for age.

**Table 1:** Baseline characteristics.

Demographics	Controls (n=14)	Patients (n=14)
DPSI, 0-4	**2.9 ± 0.3**	3.9 ± 0.2**
Mean PPD, mm	**3.7 ± 0.6**	5.0 ± 1.3**
Deepest PPD, mm	**4.6 ± 0.7**	6.4 ± 1.6**
Age, years	**54 ± 10**	63 ± 6*
Sex, % men	57 (8)	36 (5)
BMI, kg/m2	24.9 ± 3.5	28.0 ± 6.5
SBP, mmHg	130 ± 17	137 ± 17
DBP, mmHg	80 ± 7	89 ± 12
Hypertension, % (n)	43 (6)	64 (9)
Alcohol consumption, 0-8	3.3 ± 1.7	2.9 ± 1.4
Current smoking, % (n)	14 (2)	7 (1)
Smoking, packyears	**4.5 ± 5.7**	15.8 ± 18.2*
Anti-hypertensive treatment, % (n)	14 (2)	29 (4)
Lipid lowering therapy, % (n)	7 (1)	29 (4)
Fasting glucose, mmol/L	5.1 ± 0.4	5.4 ± 0.4
Creatinine, mmol/L	79 ± 12	75 ± 10
Tchol, mmol/L	4.96 ± 0.47	5.18 ± 0.76
LDLc, mmol/L	†2.65 ± 0.44	2.90 ± 0.67
HDLc, mmol/L	1.41 ± 0.47	1.44 ± 0.37
TG, mmol/L	1.92 ± 1.19	1.90 ± 0.88
nHDLc, mmol/L	†3.52 ± 0.73	3.76 ± 0.84

Demographics are reported as mean ± SD or % (number of participants) in control participants (n = 14) and participants with periodontitis (n = 14). Normally distributed data were analyzed using independent samples T-test, qi-square test was used for categorial data. Anti-hypertensive treatment includes beta-blockers, ACE-inhibitors, ARB’s and thiazide diuretics. Alcohol consumption is the sum of alcohol consumption frequency (0-4, ranging from never: 0 to >4x/week:4) and alcohol consumption quantity per occasion (0-4, ranging from 1-2 drinks:0 to >10 drinks:4) based on the Alcohol Use Disorders Identification Test questions. ^†^Data is missing for 1 participant. * indicates P < 0.05, **P < 0.01. PPD, probing pocket depth. Bold values express differences of interest, with a p-value <0.10.

#### Systemic Inflammatory Markers

Higher circulating leukocytes (median 6.1 *versus* 5.6 10^6^/mL, p<0.01) and a trend for higher lymphocytes numbers (1.8 *versus* 1.7 10^6^/mL, p=0.06) were found in patients compared to controls after age correction. In addition, a tendency for higher concentrations of circulating pro-inflammatory IL-6 and anti-inflammatory IL-1Ra cytokines were observed, but after age correction the statistical significance was lost (IL-6: 2.0 *versus* 1.4 pg/mL, p=0.08; IL-1Ra: 247 *versus* 171 pg/mL, p=0.10) (**Table 2**).

**Table 2:** Circulating cell types and cytokines/chemokines.

Cell types	Controls	Patients
WBC, 10^6^/mL	**5.6 [4.5-6.2]**	6.1 [5.5-7.1]**
Neutrophils, 10^6^/mL	3.3 [2.7-3.8]	3.2 [3.0-4.3]
Lymphocytes, 10^6^/mL	**1.7 [1.5-2.1]**	**1.9 [1.6-2.2]^**
Monocytes, 10^6^/mL	0.4 [0.3-0.5]	0.5 [0.4-0.6]
Monocytes, %	7.1 [6.1-8.5]	7.1 [6.5-8.5]
Eosinophils, 10^6^/mL	0.14 [0.08-0.19]	0.10 [0.07-0.15]
Basophils, 10^6^/mL	0.03 [0.02-0.04]	0.04 [0.03-0.05]
Classical monocytes, %gated	82 [75-84]	80 [75-85]
Intermediate monocytes, %gated	8.2 [6.3-10]	9.0 [7.0-13]
Nonclassical monocytes, %gated	10 [9.4-14]	10 [8-14]
CCR2+ monocytes, %gated	84 [80-88]	86 [81-89]
CD11b+ monocytes, %gated	87 [67-96]	91 [88-94]
CD41+ monocytes, %gated	7.4 [6.0-9.28]	7.0 [6.0-9.5]
**Cytokines & chemokines**		
IL-1β, pg/mL	0.17 [0.14-0.24]	0.17 [0.13-0.21]
IL-1Ra, pg/mL	**171 [145-239]**	**247 [188-428]^**
IL-6, pg/mL	**1.4 [0.9-1.9]**	**2.0 [1.6-3.0]^**
IL-18, pg/mL	159 [120-189]	182 [136-244]
hsCRP, µg/mL	0.7 [0.3-1.8]	0.7 [0.4-1.3]

Circulating cells/markers are reported as median with [interquartile ranges] in control participants (n = 14) and participants with periodontitis (n = 14). 10 samples under IL-1β lowest detection limit of 0.16 pg/mL. One sample under IL-6 lowest detection limit of 0.70 pg/mL. P-values are age corrected using ANCOVA. ^ indicates P < 0.10, for lymphocytes p = 0.06, IL-6: p = 0.08, IL-1Ra: p = 0.10. **P < 0.01. WBC: white blood cell counts. Bold values express differences of interest, with a p-value <0.10.

**Figure 3:**
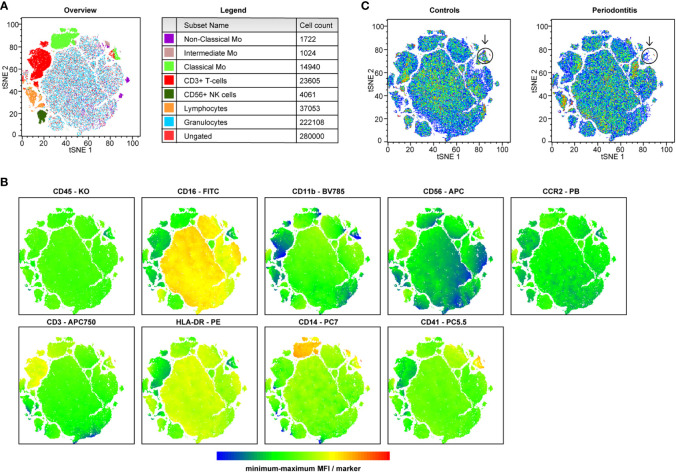
Unsupervised t-Stochastic Neighbor Embedding (tSNE) analysis. Unsupervised tSNE plots show the contribution of each expression marker in each cell type. **(A)** The overview presents the identified circulating cell types, see the legend within the table. **(B)** The distribution of each expression marker per cell type. The color depicts the MFI expression of each marker (blue: low MFI, red: high MFI). **(C)** The difference between control participants (n=14) and participants with periodontitis (n=14) is shown for each cell type. The arrow indicates a visual smaller group of platelet-lymphocyte complexes in the periodontitis’ group compared to the control group.

Using flow cytometry, circulating immune cell populations were further identified based on their expression markers (**Figure 3**). Unsupervised t-Stochastic Neighbor Embedding (tSNE) analysis showed a large overlap between both groups with potentially interesting differences in cell populations (**Figure 3B**). The most distinct population was a smaller cluster of T-cells with high CD41 expression in patients compared to controls (**Figure 3C**, arrows). Further analysis of this lymphocyte-platelet interacting cell population was not allowed as the flow cytometry panel used was designed for monocyte activation analysis and did not include further T-cell markers. Manual gating of these populations and statistical analysis also revealed high similarity between groups (**Table 2**).

#### Cytokine Production Capacity

No significant difference in the cytokine production capacity of PBMCs between patients and controls was observed (**Table 3**), for both 24h stimulation with LPS or P3C stimulation (*i.e.* innate immune response) and 7 day stimulation with whole pathogens *C. albicans* or *S. aureus* (*i.e.* adaptive immune response).

**Table 3:** Cytokine production capacity.

24-hour stimulation	Controls	Patients
LPS stimulated PBMCs		
TNFα, pg/mL	637 [414-1204]	324 [212-976]
IL-1β, pg/mL	5222 [3893-11317]	6950 [4808-11264]
IL-6, ng/mL	13.1 [7.9-15.8]	12.0 [6.9-20.8]
IL-8, ng/mL	107 [89-123]	110 [68-139]
IL-10, pg/mL	182 [91-295]	110 [59-236]
P3C stimulated PBMCs		
TNFα, pg/mL	557 [88-1338]	338 [145-681]
IL-1β, pg/mL	4216 [3112-5929]	5230 [3982-6720]
IL-6, ng/mL	13.2 [11.3-17.4]	13.6 [10.8-17.6]
IL-8, ng/mL	148 [129-189]	146 [126-185]
IL-10, pg/mL	47 [47-131]	55 [47-97]
7-day stimulation		
***C. albicans* stimulated PBMCs**		
IFNγ, pg/mL	1500 [727-1500]	1276 [625-1500]
IL-17, pg/mL	1509 [631-2811]	1033 [575-2137]
IL-22, pg/mL	5855 [2723-9233]	3803 [1120-9906]
*S. aureus* stimulated PBMCs		
IFNγ, pg/mL	1500 [604-1500]	1040 [508-1500]
IL-17, pg/mL	171 [39-401]	77 [39-127]
IL-22, pg/mL	472 [108-661]	189 [85-787]

Cytokine production capacity of PBMCs in control participants (n = 14) and participants with periodontitis (n = 14) after 24h stimulation with LPS or P3C to assess the innate immune response, and 7d stimulation with C. albicans or S. aureus to assess the adaptive immune response. Data are reported as median with [interquartile ranges], p-values are age corrected using ANCOVA.

### Periodontal, Hematopoietic, and Arterial Wall Activation on [^18^F]FDG PET/CT

**Figure 4:**
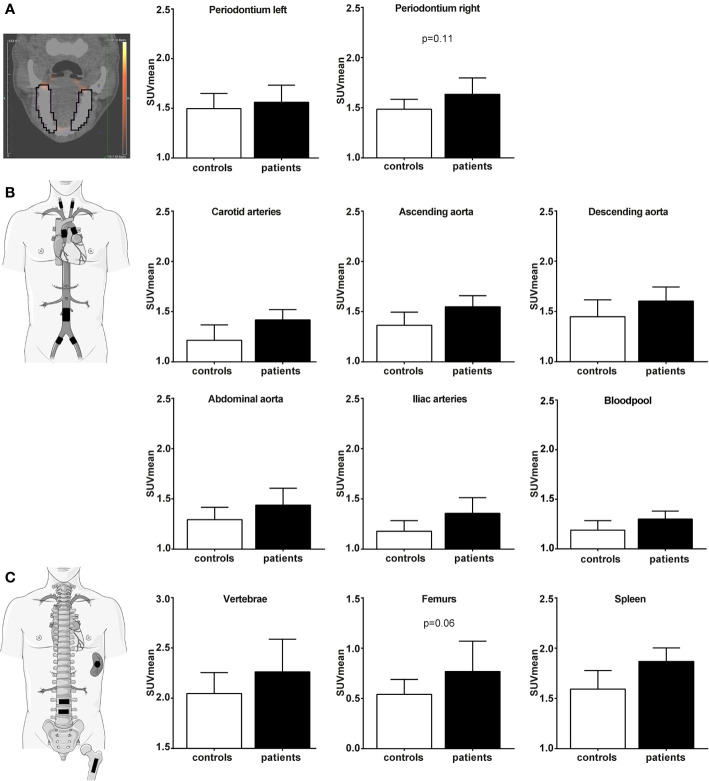
Periodontal, vascular wall inflammation and hematopoietic tissue activation on [^18^F]FDG PET/CT scan. Standard uptake value (SUV) of each region in control participants (white bars, n=14) and participants with periodontitis (black bars, n=14). Regions of interest are illustrated in the figure with black lines/blocks. **(A)** Periodontal [^18^F]FDG-uptake (SUV_mean_), **(B)** Vascular wall [^18^F]FDG-uptake (SUV_mean_), and **(C)** Hematopoietic [^18^F]FDG-uptake (SUV_mean_). Raw data is shown as geometric mean with 95% CI, and p-values are age corrected with ANCOVA.

[^18^F]FDG PET/CT imaging results are discussed before (*i.e.* raw data) and after correction for age. The figures display raw data with age-corrected p-values. Increased [^18^F]FDG-uptake in periodontium was observed in patients compared to controls (SUV_mean_ 1.70 *versus* 1.49, p<0.05). After age correction, the statistical significance was diminished (**Figure 4A**, p=0.11). The SUV in ascending aorta, carotid and iliac arteries and main hematopoietic regions were higher in patients, including the blood pool used to calculate target-to-background ratios (TBR) (**Appendix Table 2**). Age correction resulted in the loss of statistical significance for arterial wall [^18^F]FDG-uptake (**Figure 4B**). A trend for higher [^18^F]FDG-uptake in hematopoietic tissue of patients remained for the femur bone marrow (SUV_mean_ 0.88 *versus* 0.58, p=0.06) (**Figure 4C**).

The TBR values showed no statistically significant difference using uncorrected p-values between patients and controls. Only a trend for femur bone marrow activation was observed in patients after age correction (TBR_mean_ 0.40 *versus* 0.31, p=0.08) (**Appendix Table 3**).

### Periodontitis Severity Correlates With Circulating Markers and Tissue Inflammation

**Figure 5:**
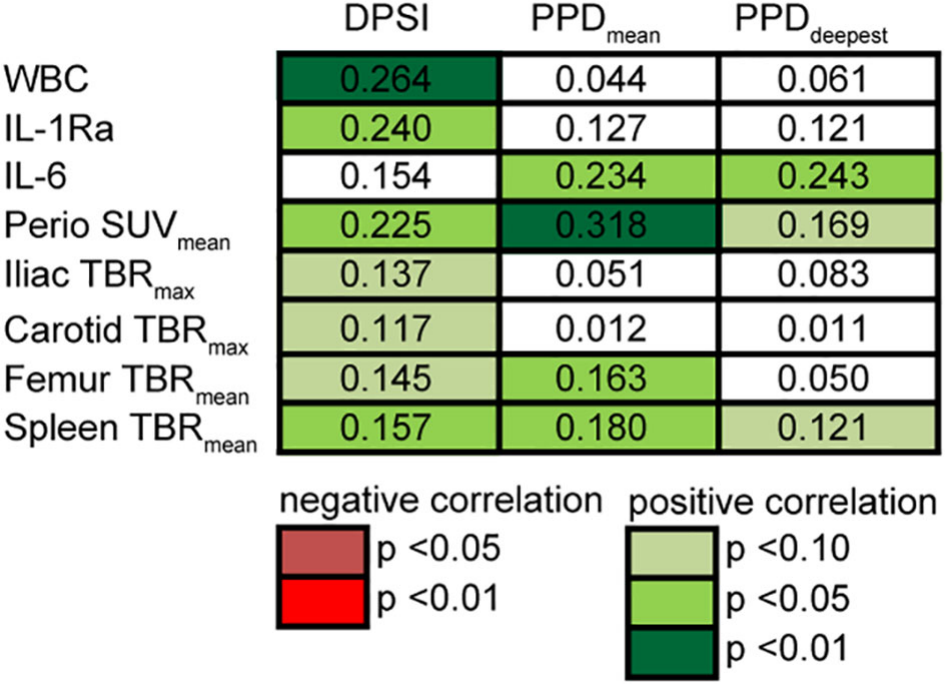
Correlation of periodontitis severity with outcomes. Associations of DPSI score (0–4) and mean and deepest PPD (mm) with circulating markers and periodontal, vascular, hematopoietic [^18^F]FDG-uptake shows strong associations. Linear regression analyses on log-transformed data accounting for age (n = 28). Multiple correlation coefficient R^2^ of regression model noted in cells. P-values of each dependent variable are displayed by color.

The severity of PD, either measured by DPSI or PPD, showed strong positive associations with circulating markers IL-6 and IL-1Ra, and with iliac artery vascular wall and femur hematopoietic tissue [^18^F]FDG-uptake (**Figure 5** and **Appendix Table 4**). For example, the DPSI score strongly correlated with the number of circulating leukocytes (p<0.01) and mildly correlated with vascular wall inflammation in the iliac arteries (p=0.10). Also the mean PPD associated with circulating IL-6 concentrations (p<0.05), periodontal tissue inflammation (p<0.01) and splenic tissue activity (p<0.05). For circulating markers IL-1β, IL-18, CRP, circulating cell types, and other vascular regions no association with PD was found.

## Discussion

Our aim was to explore the induction of trained immunity as a plausible mechanistic link between PD and atherosclerosis. *In vitro*, we showed that *P. gingivalis* induces trained immunity in human monocytes, in terms of an augmented cytokine production capacity. *In vivo*, patients with severe PD had signs of systemic inflammation, with higher circulating leukocyte counts. However, their circulating monocytes did not show a hyperresponsive phenotype. Patients appeared to have periodontal and hematopoietic tissue activation as visualized by [^18^F]FDG PET/CT, although this was not significant after age correction, which correlated with PD severity.

First, we set up an *in vitro* study using a validated trained immunity protocol (14). Previously we showed that exposure of human monocytes to microbial stimuli, including *Candida albicans* and Bacille Calmette-Guérin (BCG), and endogenous atherogenic stimuli, including oxLDL and catecholamines, leads to an augmented cytokine production in response to restimulation (27, 28) (13, 29, 30). In the current study, two different *P. gingivalis* strains were also capable of inducing trained immunity. Two *P. gingivalis* strains with different virulent rates, based on its capacity to invade endothelial cells (10), were chosen to investigate the trainings effect in healthy donors. Interestingly, the more virulent W83 strain induced a stronger trained immunity response than ATCC. The augmented cytokine production was most pronounced after restimulation with P3C, a TLR2 agonist, compared to LPS, a TLR4 agonist. This supports the reports that *P. gingivalis* drives a chronic inflammatory response *via* activation of TLR2 and inhibition of TLR4 in immune cells (7). TLR2-mediated activation is relevant in the context of pathogen induced atheroma formation in mice (31). The induced two-fold increase in IL-6 and TNFα production by *P. gingivalis* was comparable but lower compared to the effect size of oxLDL (see **Figure 1D** for oxLDL training) and BCG (13, 28). *P. gingivalis* did not significantly induce foam cell formation in macrophages, possibly due to the sample size. In mice, direct stimulation with *P. gingivalis* did increase foam cell formation (32). The PBMCs used for the *in vitro* experiments were obtained from healthy blood donors. Their periodontitis status and cardiovascular risk factors are unknown which could potentially act as confounding factors. Nevertheless, despite the possible confounders of the donors, we found an significant trained immunity response to *P. gingivalis*. Future experiments should elucidate the cellular mechanisms by which *P. gingivalis* induces trained immunity. Together this suggests that trained immunity might fuel a self-perpetuating chronic inflammation in the periodontium.

Our second aim was to translate our *in vitro* findings to patients with periodontitis *in vivo*. In this study, participants were included with PD based on validated clinical index and radiographs. Although we aimed to match the patient and control group, age was significantly different and subsequently all results were corrected for age. In this exploratory study, we presented our data before (*i.e.* raw data) and after age correction. In contrast to the *in vitro* results of PG-induced trained immunity, no difference in the cytokine production capacity of circulating monocytes, which is regarded as major indicator of trained immunity, between individuals with severe PD and those without was observed. This is in contrast to previous studies that did show trained immunity characteristics *in vivo*: after BCG vaccination *in vivo*, isolated monocytes show a hyperresponsive trained phenotype for several months after vaccination (28). Also, isolated monocytes from subjects with dyslipidemia or pheochromocytoma show a trained phenotype, matching the *in vitro* observations that oxLDL and catecholamines induce trained immunity (33). There are various explanations for this discrepancy between the *in vitro* and *in vivo* findings in the current study. First, in comparison to BCG vaccination and pheochromocytoma that have systemic effects, PD is a local disease which might not influence the phenotype of circulating immune cells. Secondly, the disease severity in our patient group might not be severe enough to exert a significant effect on distant immune cells. Thirdly, the *in vitro* training effect of *P. gingivalis* might be diluted *in vivo* by influences of other stimuli. Our results do not exclude, however, that trained immunity by *P. gingivalis* occurs locally in the inflamed periodontium, or within atherosclerotic plaques.

It is worth to emphasize that our patients with severe PD had signs of systemic inflammation, with significantly higher circulating leukocytes, supporting the existing evidence for the presence of systemic inflammation in PD (2). In detail, tSNE analysis revealed a lower subset of T-lymphocytes with platelet interaction in PD. The role of lymphocytes in the development of periodontitis in an interesting topic for future research. To test the hypothesis that the mechanism of trained immunity could contribute to the development of CVD by augmenting the inflammatory component of atherosclerosis in patients with periodontitis, we assessed vascular wall inflammation by PET-CT scanning. A trend for periodontal and hematopoietic tissue activation was observed. Moreover, positive correlations between the severity of PD and circulating inflammatory markers or vascular wall inflammation were found. The mild association with vascular inflammation was only observed in iliac arteries, which is a region that is prone for early atherosclerotic plaque development. There are only a few studies using PET-CT scanning that show that periodontal [^18^F]FDG-uptake correlates with vascular wall inflammation (34), and that lipid-lowering therapy significantly decreases vascular wall inflammation as well as periodontal inflammation (35). However, major limitations of these studies are that participants were retrospectively selected from other cohorts without clinical diagnosis of PD.

### Limitations of the Study

What makes our study unique is that key factors involved in the etiology and pathogenesis of PD, including *P. gingivalis* as keystone pathogen and the host innate immune response, were investigated together, in detail, and prospective in relation to atherosclerotic inflammation. Highly sensitive methods for evaluation of innate immune cells were used, and [^18^F]FDG PET/CT scanning was applied for bone marrow, periodontal and vascular wall inflammation. Limitations are the small sample size (n=14 per group) and that participants with severe PD were in supportive periodontal therapy, which resulted in a milder phenotype. It cannot be excluded this influenced our results, as periodontal therapy can reduce surrogate markers of CVD (2). Together, this may explain the lack of differences in cytokine production capacity and vascular wall inflammation. Larger cross-sectional studies with untreated cases of severe PD are needed to further explore trained immunity in this population.

## Data Availability Statement

The raw data supporting the conclusions of this article will be made available by the authors, without undue reservation.

## Ethics Statement

The studies involving human participants were reviewed and approved by Institutional Review Board Arnhem/Nijmegen, the Netherlands (NL61840.091.17). The patients/participants provided their written informed consent to participate in this study.

## Author Contributions

MPN contributed to conception, design, data acquisition and interpretation, performed all statistical analyses, drafted and critically revised the manuscript. AP and NR contributed to conception, design, data acquisition and interpretation, drafted and critically revised the manuscript. ES, EA, PV, and SB contributed to data acquisition and interpretation, and critically revised the manuscript. LJ and MGN contributed to conception, design, and critically revised the manuscript. All authors contributed to the article and approved the submitted version.

## Funding

This work was supported by the European Union’s Horizon 2020 research and innovation program [grant number No 667837] and by the Dutch Heart Foundation IN-CONTROL CVON grant [CVON2018-27] to [MGN, LJ, NR]; Netherlands Organization for Scientific Research Spinoza Grant [NWO SPI 94-212] to [MGN]; ERC Advanced Grant (#833247) to [MGN]; Competitiveness Operational Programme grant of the Romanian Ministry of European Funds [HINT, ID P_37_762; MySMIS 103587] to [LJ]; the ERA-CVD Joint Transnational Call 2018 by the Dutch Heart Foundation [JTC2018, project MEMORY; 2018T093] to [NR].

## Conflict of Interest

LJ and MGN are scientific founders of TTxD.

The remaining authors declare that the research was conducted in the absence of any commercial or financial relationships that could be construed as a potential conflict of interest.

## Publisher’s Note

All claims expressed in this article are solely those of the authors and do not necessarily represent those of their affiliated organizations, or those of the publisher, the editors and the reviewers. Any product that may be evaluated in this article, or claim that may be made by its manufacturer, is not guaranteed or endorsed by the publisher.
