# Supplementary material for: An Explorative Study on Monocyte Reprogramming in the Context of Periodontitis In Vitro and In Vivo
Source: Front Immunol. 2021 Aug 13;12:695227. doi: 10.3389/fimmu.2021.695227 (PMC8414567; doi:10.3389/fimmu.2021.695227)
Supplement: Supplementary file 1 [file DataSheet_1.docx]

**An explorative study on monocyte reprogramming in the context of periodontitis *in vitro* and *in vivo***

**Supplementary Methods**

[^18^F]FDG PET and low-dose CT scanning

Participants underwent [^18^F]FDG PET with low-dose non-contrast enhanced CT from skull base to the trochanter major on a dedicated Siemens Biograph 40 mCT scanner (Siemens Healthineers, Erlangen, Germany). After adhering to a 24-hour low-carbohydrate diet and 6 hours of fasting, ~2.1 MBq/kg [^18^F]FDG was administered intravenously, as described previously (1). Glucose concentrations were obtained (5.3±0.5 mmol/L) after injection. Before scanning, participants rested in supine position for 2 hours.

Images were reconstructed according to EARL protocols; using a TrueX algorithm with point spread function (PSF) and time-of-flight (TOF) measurements, using 3 iterations, 21 subsets, matrix size 200x200 (pixel spacing of 4.07 mm), Full Width Half Maximum (FWHM) of 3 mm and using 2 minutes of PET data. Postprocessing was performed using a 3D Gaussian filter kernel, 3.0 mm, using the Inveon Research Workspace 4.2 (Preclinical Solutions, Siemens Medical Solutions USA, Knoxville, USA).

Periodontal [^18^F]FDG-uptake was determined in two volumes of interest (VOI) by two independent operators [MPN, AP]: the left and right periodontal tissue. Anatomic landmarks, the foramen mentale in the mandible and the sinus maxillaris in the upper jaw, were used to identify the periodontal regions of interest (ROI). Next, four ROIs were drawn on the transversal slices (2 lower jaw, 2 upper jaw on each side) in the molar/premolar area based on CT imaging. By auto-interpolation of the layers the selected ROI became volume of interest (VOI) (Appendix Figure 2). [^18^F]FDG-uptake in the vascular wall was determined in seven ROI; the aorta ascendens, aorta descendens, abdominal aorta, the left and right common carotid arteries, and the left and right iliac arteries. ROIs were positioned in uncalcified areas of vascular wall. [^18^F]FDG-uptake in haematopoietic tissue was assessed in the spleen, lumbar vertebrae L2 and L3, and in the left and right medullary cavity of the femur. These regions of interest were evaluated using the Inveon Research Workspace 4.2.

Operators that determined the VOIs/ROIs were blinded for all participants’ characteristics (including control or patient). The standardized uptake value (SUV) was extracted from each ROI after correction for [^18^F]FDG dose (MBq) and bodyweight using the PyRadiomics toolbox (2). The SUVs of left and right ROIs (*e.g.* left and right carotid artery) were averaged. Next, the target-to-background ratio (TBR) was calculated as the ratio of the vascular wall SUV and the mean thoracic arterial blood pool SUV. The TBRs of haematopoietic tissue were expressed as ratio of the mean liver SUV, as recommended by the European guideline (1).

**Supplementary Tables and Figures**

Appendix Table 1. Cytokine production after restimulation

| Condition | Control | Pg W83 | Pg ATCC |
| --- | --- | --- | --- |
| LPS restimulated monocytes |  |  |  |
| TNFα, pg/mL | **672 ±94** | **790 ±96^** | **1010 ±224^** |
| IL-6, ng/mL | **799 ±96** | **1042 ±113**** | **1056 ±142*** |
| P3C restimulated monocytes |  |  |  |
| TNFα, pg/mL | **190 ±27** | **321 ±51**** | **356 ±78**** |
| IL-6, ng/mL | **787 ±109** | **1173 ±137**** | **1159 ±158*** |

Absolute cytokine concentrations after training with *P. gingivalis* as shown in Figure 1B. Mean with SEM, Wilcoxon signed rank-test. **: P-value <0.01, *: P-value <0.05, ^: P-value <0.10. Pg: *P. gingivalis.*

Appendix Table 2. Standard uptake values of [^18^F]FDG PET/CT scan.

| Regions of interest | Controls | Patients |
| --- | --- | --- |
| Carotid arteries, SUV_mean_ | **1.24 ±0.23** | **1.43 ±0.17*** |
| Carotid arteries, SUV_max_ | **1.53 ±0.29** | **1.80 ±0.28*** |
| Ascending aorta, SUV_mean_ | **1.38 ±0.21** | **1.56 ±0.19*** |
| Ascending aorta, SUV_max_ | 2.04 ±0.36 | 2.22 ±0.32 |
| Descending aorta, SUV_mean_ | 1.47 ±0.26 | 1.62 ±0.24 |
| Descending aorta, SUV_max_ | 2.24 ±0.52 | 2.50 ±0.44 |
| Abdominal aorta, SUV_mean_ | 1.31 ±0.20 | 1.46 ±0.26 |
| Abdominal aorta, SUV_max_ | **1.82 ±0.31** | **2.08 ±0.36^** |
| Iliac arteries, SUV_mean_ | **1.25 ±0.28** | **1.38 ±0.24*** |
| Iliac arteries, SUV_max_ | **1.56 ±0.31** | **1.83 ±0.29*** |
| Blood pool, SUV_mean_ | **1.20 ±0.16** | **1.31 ±0.14^** |
| Vertebrae, SUV_mean_ | 2.07 ±0.32 | 2.32 ±0.60 |
| Vertebrae, SUV_max_ | 2.49 ±0.39 | 2.90 ±0.74 |
| Medullary cavity of femur, SUV_mean_ | **0.58 ±0.24** | **0.88 ±0.45^** |
| Medullary cavity of femur, SUV_max_ | **1.11 ±0.37** | **1.50 ±0.62^** |
| Spleen, SUV_mean_ | **1.62 ±0.29** | **1.88 ±0.23*** |
| Spleen, SUV_max_ | **1.91 ±0.34** | **2.21 ±0.34*** |

SUV values of regions of interest in control participants (n=14) and participants with periodontitis (n=14). Raw data are reported as mean ±SD, uncorrected p-values using independent samples T-test. ^ indicates P<0.10, for abdominal aorta p=0.06; blood pool p=0.06; femur max p=0.06; femur mean p =0.07. SUV: standard uptake value.

Appendix Table 3. Target-to-background values of [^18^F]FDG PET/CT scan.

| Regions of interest | Controls | Patients |
| --- | --- | --- |
| Carotid arteries, TBR_mean_ | 1.03 ±0.16 | 1.10 ±0.16 |
| Carotid arteries, TBR_max_ | 1.28 ±0.19 | 1.38 ±0.25 |
| Ascending aorta, TBR_mean_ | 1.15 ±0.12 | 1.20 ±0.13 |
| Ascending aorta, TBR_max_ | 1.71 ±0.22 | 1.71 ±0.23 |
| Descending aorta, TBR_mean_ | 1.23 ±0.15 | 1.24 ±0.15 |
| Descending aorta, TBR_max_ | 1.87 ±0.37 | 1.92 ±0.27 |
| Abdominal aorta, TBR_mean_ | 1.10 ±0.15 | 1.12 ±0.16 |
| Abdominal aorta, TBR_max_ | 1.52 ±0.23 | 1.59 ±0.23 |
| Iliac arteries, TBR_mean_ | 1.04 ±0.16 | 1.05 ±0.15 |
| Iliac arteries, TBR_max_ | 1.30 ±0.15 | 1.40 ±0.16 |
| Vertebrae, TBR_mean_ | 1.07 ±0.16 | 1.07 ±0.26 |
| Vertebrae, TBR_max_ | 1.30 ±0.19 | 1.33 ±0.33 |
| Medullary cavity of femur, TBR_mean_ | **0.31 ±0.14** | **0.40 ±0.20^** |
| Medullary cavity of femur, TBR_max_ | **0.58 ±0.21** | **0.68 ±0.26^** |
| Spleen, TBR_mean_ | 0.83 ±0.08 | 0.86 ±0.09 |
| Spleen, TBR_max_ | 0.99 ±0.09 | 1.01 ±0.13 |

TBR values of regions of interest in control participants (n=14) and participants with periodontitis (n=14). Raw data are reported as mean ±SD, p-values are age corrected using ANCOVA. ^ indicates P<0.10. TBR: target-to-background ratio.Appendix Table 4. Correlation of periodontitis severity with outcomes.

| Model +age | R^2^ | R^2^-adj | p-value (model) | Unst β | Confidence interval | p-value (variable) |
| --- | --- | --- | --- | --- | --- | --- |
| DPSI |  |  |  |  |  |  |
| WBC | **0.264** | **0.205** | **0.02** | **0.10** | **0.03-0.17** | **0.01** |
| IL-1Ra | **0.240** | **0.176** | **0.04** | **0.19** | **0.03-0.35** | **0.02** |
| IL-6 | 0.154 | 0.086 | 0.12 | 0.13 | -0.07-0.33 | 0.18 |
| Perio SUV_mean_ | **0.225** | **0.163** | **0.04** | **0.06** | **0.01-0.12** | **0.02** |
| Iliac TBR_max_ | 0.137 | 0.068 | 0.16 | 0.03 | -0.01-0.08 | 0.11 |
| Carotid TBR_max_ | **0.117** | **0.046** | **0.21** | **0.05** | **-0.01-0.11** | **0.09** |
| Femur TBR_mean_ | **0.145** | **0.077** | **0.14** | **0.16** | **-0.01-0.34** | **0.07** |
| Spleen TBR_mean_ | **0.157** | **0.090** | **0.12** | **0.03** | **0.00-0.07** | **0.05** |
| PPD_mean_ |  |  |  |  |  |  |
| WBC | 0.044 | -0.033 | 0.57 | 0.01 | -0.02-0.05 | 0.36 |
| IL-1Ra | 0.127 | 0.055 | 0.20 | 0.05 | -0.02-0.11 | 0.17 |
| IL-6 | **0.234** | **0.172** | **0.04** | **0.08** | **0.00-0.16** | **0.04** |
| Perio SUV_mean_ | **0.318** | **0.264** | **0.01** | **0.03** | **0.01-0.05** | **0.01** |
| Iliac TBR_max_ | 0.051 | -0.025 | 0.52 | 0.01 | -0.01-0.02 | 0.66 |
| Carotid TBR_max_ | 0.012 | -0.067 | 0.86 | 0.01 | -0.02-0.03 | 0.85 |
| Femur TBR_mean_ | **0.163** | **0.097** | **0.11** | **0.07** | **0.00-0.14** | **0.05** |
| Spleen TBR_mean_ | **0.180** | **0.114** | **0.08** | **0.02** | **0.00-0.03** | **0.03** |
| PPD_deep_ |  |  |  |  |  |  |
| WBC | 0.061 | -0.014 | 0.45 | 0.01 | -0.01-0.04 | 0.26 |
| IL-1Ra | 0.121 | 0.048 | 0.21 | 0.04 | -0.02-0.09 | 0.19 |
| IL-6 | **0.243** | **0.182** | **0.03** | **0.07** | **0.01-0.13** | **0.03** |
| Perio SUV_mean_ | **0.169** | **0.103** | **0.10** | **0.02** | **0.00-0.04** | **0.06** |
| Iliac TBR_max_ | 0.083 | 0.010 | 0.34 | 0.01 | -0.00-0.02 | 0.31 |
| Carotid TBR_max_ | 0.011 | -0.068 | 0.87 | -0.01 | -0.02-0.02 | 0.86 |
| Femur TBR_mean_ | 0.050 | -0.026 | 0.53 | 0.03 | -0.04-0.09 | 0.39 |
| Spleen TBR_mean_ | **0.121** | **0.051** | **0.20** | **0.01** | **0.00-0.02** | **0.09** |

Associations of DPSI score (0-4) and mean and deepest PPD (mm) with circulating markers and periodontal, vascular, hematopoietic [^18^F]FDG-uptake shows strong associations. Linear regression analyses on log-transformed data accounting for age (n=28).

Appendix Figure 1. Gating strategy of circulating cells.


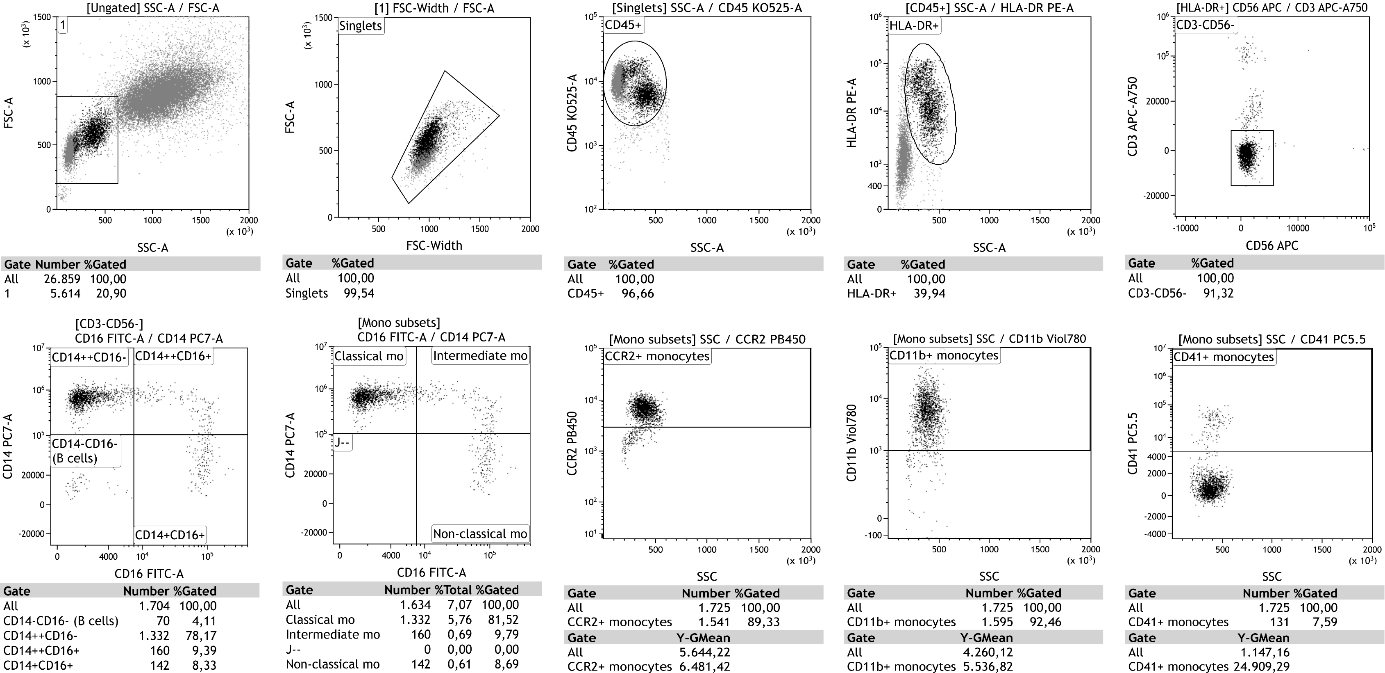


Monocytes were selected based on CD45+ HLA-DR+ and monocyte scatter properties, then CD3+ T-lymphocytes and CD56+ NK-cells were excluded, and monocyte subsets were identified in the CD14/CD16 plot as percentage of gated.

Appendix Figure 2. Volume of interest of periodontal tissue on [^18^F]FDG PET/CT scan.


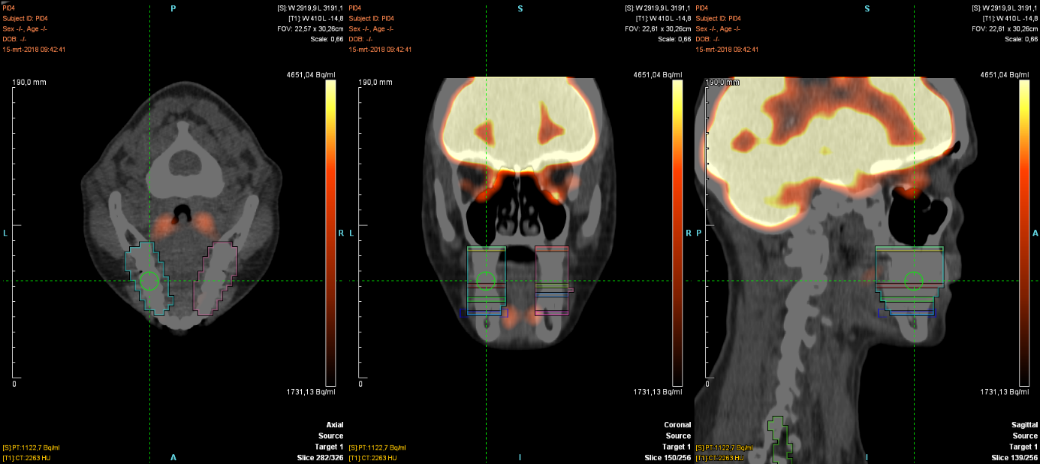


Representative figure of periodontal volume of interest based on four regions of interest drawn in the transversal slice per side.

**Supplementary references**

1. Bucerius J, Hyafil F, Verberne HJ, Slart RH, Lindner O, Sciagra R, et al. Position paper of the Cardiovascular Committee of the European Association of Nuclear Medicine (EANM) on PET imaging of atherosclerosis. Eur J Nucl Med Mol Imaging. 2016;43(4):780-92.

2. van Griethuysen JJM, Fedorov A, Parmar C, Hosny A, Aucoin N, Narayan V, et al. Computational Radiomics System to Decode the Radiographic Phenotype. Cancer Res. 2017;77(21):e104-e7.
